# Correlation of Hypothyroidism With Disease Activity Score-28 in Patients of Rheumatoid Arthritis

**DOI:** 10.7759/cureus.26382

**Published:** 2022-06-27

**Authors:** Amer Zohaib, Aflak Rasheed, Tafazzul-e-Haque Mahmud, Umar Hayat, Sumayya Shabbir, Saima Riaz, Muhammad Zahid Z Jamil, Umair Javaid

**Affiliations:** 1 Rheumatology, Shaikh Zayed Postgraduate Medical Institute, Lahore, PAK; 2 Nephrology, Jinnah Hospital, Lahore, PAK; 3 Internal Medicine, Mayo Hospital, Lahore, PAK; 4 Internal Medicine and Endocrinology, Jinnah Hospital, Lahore, PAK

**Keywords:** esr, joint inflammation, disease activity score-28, hypothyroidism, rheumatoid arthritis

## Abstract

Introduction

Rheumatoid arthritis (RA) is a chronic autoimmune disorder with variable disease course including periods of flares and remissions. High disease activity in terms of disease activity score-28 (DAS-28) results in significant morbidity. Hypothyroidism is found to be associated with higher DAS-28 scores in RA. This study is planned to determine overt and subclinical hypothyroidism and its correlation with the DAS-28 score in patients with RA.

Methodology

This study was conducted from June 2021 to March 2022 at the department of rheumatology and immunology at Shaikh Zayed Hospital, Lahore, Pakistan. Inclusion criteria were any male and female patients aged between 18 and 70 years. The blood samples of diagnosed patients with RA were sent for thyroid function tests (thyroxine [FT4], thyroid-stimulating hormone [TSH]), and erythrocyte sedimentation rate (ESR), and the patients were categorized as overt hypothyroidism, subclinical hypothyroidism, and non-hypothyroid. The collected data were analyzed on Statistical Package for the Social Sciences (SPSS) version 24.0 (IBM Corp., Armonk, NY).

Results

The mean age of patients was 38.18 ± 9.78 years. The mean duration of symptoms was 14.65 ± 1.04 months. There were 182 (91%) females and 18 (9%) males. The mean number of swollen joints was 2.26 ± 2.8, and the mean number of tender joints was 4.16 ± 5.11. Sixty patients (30%) had high disease activity, i.e., DAS-28 score > 5.1. Fifty-seven patients (28.5%) with RA had subclinical hypothyroidism, and 19 patients (9.5%) had overt hypothyroidism. Pain visual analog scale (VAS) and DAS-28 were significantly higher in hypothyroid patients.

Conclusion

It was concluded that patients of RA with concomitant hypothyroidism had increased disease activity with increased tender joints. Thyroid function tests should be included in the clinical evaluation of RA patients. The evaluation of thyroid functional status must be done during screening in RA patients. This will detect thyroid disorders earlier, with early treatment initiation and possibly a better prognosis.

## Introduction

Rheumatoid arthritis (RA) is a chronic autoimmune disorder involving multiple joints, which results in their inflammation and destruction, causing various disabilities and impaired mobility [1]. The incidence of RA in the general population is 0.5%-1%. The mechanism causing RA is still under investigation. The proposed mechanism is a complex interaction between environmental and genetic factors that result in activation of the response generated by the immune system and synovial inflammation in a discrete symmetric outline [2]. Along with its clinical manifestations, patients with RA are at an increased risk of developing various types of carcinomas, cardiovascular disease (CVD), diabetes mellitus, vasculitis, lung diseases, deafness, and psychiatric illnesses, which are attributed to the inflammatory condition and autoimmunity of rheumatoid arthritis [3-7]. A long-term prognosis of the disease can be affected by these comorbid conditions leading to decreased quality and life span [3]. Multiple comorbidities besides their clinical manifestations can complicate the disease course [3]. In patients with RA, 6%-34% have shown the presence of thyroid dysfunction irrespective of its autoimmune origin [2].

On the contrary, if thyroid antibodies are present, the prevalence rate can rise up to about 38% [8]. In a study carried out in India in 2018 among 250 patients with RA, 41.6% of patients had hypothyroidism, subclinical and overt both combined [9]. Routine screening of thyroid dysfunction in the general population is not suggested; however, evaluation of high-risk groups is advised. These include patients having symptoms suggestive of hyperthyroidism or hypothyroidism, patients previously diagnosed patients with thyroid dysfunctions, and patients having a family history of thyroid disease and autoimmunity [2]. Specifically, in patients with RA, the presence of hypothyroidism complicates the course of RA being associated with poor initial treatment response [2]. It has been observed that RA patients with hypothyroidism have an aggressive course of the disease. According to a study, 95% of RA patients having thyroid dysfunction had high disease activity as compared to only 37% of patients without thyroid dysfunction. Due to these findings, the screening of thyroid dysfunction is recommended at the start of the treatment and then on yearly basis [9].

The prevalence of hypothyroidism and subclinical hypothyroidism in the general population of Pakistan is 4.1% and 5.4%, respectively [10]. There is limited data regarding the prevalence of hypothyroidism among RA patients in the Pakistani population, neither an association of it with disease activity has been observed in our local population. The current study is aimed to correlate hypothyroidism with the activity of the disease in previously diagnosed RA patients in our local population.

## Materials and methods

This cross-sectional study was conducted from June 2021 to March 2022 at the department of rheumatology and immunology at Shaikh Zayed Hospital, Lahore, Pakistan, after approval from the Institutional Review Board of Shaikh Zayed Medical Complex Lahore, Pakistan. The sample size of 200 RA patients was estimated using a 95% confidence level and a 5% margin of error with the expected frequency of subclinical and overt hypothyroidism as 41.6% [9]. Inclusion criteria were any male and female patients aged between 18 and 70 years. Patients diagnosed with RA according to American College of Rheumatology (ACR) criteria 2010 [11]. Exclusion criteria were pregnancy, hepatitis B or C, hypersensitivity to disease-modifying antirheumatic drugs (DMARDS), chronic kidney disease (CKD), known thyroid disease, and overlap with other rheumatological diseases like systemic lupus erythematosus (SLE), systemic sclerosis, mixed connective tissue disease (MCTD), and dermatomyositis. Non-probability consecutive sampling technique was used to include the patients. Informed consent was obtained from those fulfilling the eligibility criteria. 

From each diagnosed patient with rheumatoid arthritis, 5 cc of blood was taken and sent for thyroid function tests (thyroxine [FT4], thyroid-stimulating hormone [TSH]) and erythrocyte sedimentation rate (ESR). Blood samples were sent to the pathology laboratory of Shaikh Zayed Hospital, Lahore. The chemiluminescence immunoassay technique was used for assessing thyroid function tests. According to lab results, the patients were categorized as overt hypothyroidism, subclinical hypothyroidism, and non-hypothyroid. Diagnoses of these thyroid disorder subgroups were made according to the updated guidelines [12]. Overt hypothyroidism was labeled when TSH > 4.2 mIU/L and FT4 < 0.8 ng/dl, and subclinical hypothyroidism was labeled when FT4 was in the normal range (0.8-1.7) ng/dl. The assessment of disease activity was done by a physician, using a standardized 100-mm visual analog scale (VAS) with 0 for no activity and 100 for maximum activity. Examination of joints was done recording the number of tender and swollen joints. DAS-28-ESR was calculated and noted. DAS-28 score was interpreted as follows: DAS-28 > 5.1 indicates high disease activity, DAS-28 = 3.2-5.1 indicates moderate disease activity, DAS-28 = 2.6-3.1 indicates low disease activity, and DAS-28 < 2.6 indicates remission. Patients who had thyroid dysfunction were referred to the medical outpatient department (OPD) for management of thyroid dysfunction. Patients were divided into two groups, i.e., patients with hypothyroidism were placed in group A, and euthyroid and hyperthyroid patients were placed in group B.

The collected data was analyzed using the Statistical Package for the Social Sciences (SPSS) version 24.0 (IBM Corp., Armonk, NY). Continuous variables, age, duration of symptoms, and ESR were expressed as Mean ± SD, and categorical variables such as gender, and disease activity status on DAS-28 were summarized as frequency and percentages. To determine the association between various categorical variables, the chi-square test and Fisher’s exact test were used. Statistical analysis of means was done by independent sample test between two groups. P-value ≤ 0.05 was taken as significant.

## Results

A total of 235 patients were included who met the inclusion criteria. Among these, 35 patients who lost the follow-up were excluded. The mean age of patients was 38.18 ± 9.78 years. The mean duration of symptoms was 14.65 ± 1.04 months. There were 182 (91%) females and 18 (9%) males as shown in Table 1.

**Table 1:** Demographics of patients

	Study Group
Number of patients	200
Age (years)	38.18 ± 9.78
Gender	
Male	18 (9%)
Female	182 (91%)
Mean duration of symptoms (months)	14.65 ± 1.04

On presentation in OPD, the mean number of swollen joints was 2.26 ± 2.8, and the mean number of tender joints was 4.16 ± 5.11. The mean ESR value on presentation was 36.32 ± 21.05 mm/h. On presentation, 80 patients (40%) had severe joint pains and 60 patients (30%) had high disease activity, i.e., DAS-28 score > 5.1. It was seen that 57 patients (28.5%) with RA had newly diagnosed subclinical hypothyroidism, and 19 patients (9.5%) had newly diagnosed overt hypothyroidism. All labs and patients' DAS-28 and VAS calculated in the OPD are stated in Table 2.

**Table 2:** Clinical presentation and lab values of patients ESR: Erythrocyte sedimentation rate; VAS: Visual analog scale; DAS-28: Disease activity score-28.

		Study Group
Number of swollen joints		2.26 ± 2.8
Number of tender joints		4.16 ± 5.11
Mean ESR		36.32 ± 21.05
VAS	Mild	68 (34%)
	Moderate	52 (26%)
	Severe	80 (40%)
DAS-28 (disease activity)	Remission	28 (14%)
	Low	36 (18%)
	Moderate	76 (38%)
	High	60 (30%)
Hypothyroidism	Subclinical	57 (28.5%)
	Overt	19 (9.5%)
Euthyroidism	-	115 (57.5%)
Hyperthyroidism	-	9 (4.5%)

A total of 76 patients with overt and subclinical hypothyroidism were placed in group A and 124 euthyroid and hyperthyroid patients were placed in group B, and both groups were compared. Fifty-nine patients (77.6%) of group A had tender joints at presentation, while in group B, 37 patients (29.8%) had tender joints on presentation, which was statistically significant. Similarly, 54 patients (71.05%) of group A and 34 patients (27.41%) of group B had swollen joints on presentation, which was statistically significant. VAS and DAS-28 were significantly higher in group A when compared with group B. Comparison of group A and group B is stated in Table 3.

**Table 3:** Comparison of group A and group B Group A: RA patients with hypothyroidism; Group B: RA patients with euthyroid or hyperthyroidism; VAS: Visual analog scale; DAS-28: Disease activity score-28.

Variables		Study Group		P-value
		Group A (hypothyroid)	Group B (euthyroid + hyperthyroid)	
Number of patients having tender joints		59 (77.6%)	37 (29.8%)	0.002
Number of patients having swollen joints		54 (71.05%)	34 (27.41%)	0.00
VAS	Mild	14	94	0.001
	Moderate	23	14	
	Severe	37	19	
DAS-28	Remission	01	27	0.001
	Low	02	34	
	Moderate	36	40	
	High	37	23	

## Discussion

The discussion of thyroid disease association with RA is a matter of debate as literature shows controversial results in different studies. The association between autoimmune thyroid disease and RA is probably due to the natural course of autoimmune diseases and their tendency to overlap [13]. Environmental factors and genetic predisposition play a combined role in the development of thyroid disease and RA [14]. New identification of loci and the number of susceptible genes responsible for the development of RA and thyroid disease is in progress to identify the exact nature of diseases [15]. Due to the overlapping nature of diseases, it is difficult to clinically differentiate patients with thyroid disorders who presented with RA [16].

In our study, it was seen that 57 patients (28.5%) with RA had subclinical hypothyroidism and 19 patients (9.5%) had overt hypothyroidism. These results are comparable to a study conducted by Elattar et al., which shows that hypothyroidism was present in 36 RA patients (24%) and subclinical hypothyroidism in six patients (4%) [13]. There was a significant positive correlation found between TSH levels and RA disease activity parameters in their study [13]. Similarly, a study by Mahagna et al. found that thyroid dysfunction diseases were found in 16.0% of patients with RA [17]. Similarly, a study by Li et al. showed hypothyroidism among 26.2% of patients with RA, which presented a significant association between hypothyroidism and RA [1]. A study by Waseem et al. found in their study that 38.2% of patients with RA had hypothyroidism, which was statistically significant [9].

In our study, there were 182 (91%) females and 18 (9%) males, which shows an increasing trend of thyroid disease in the female gender. Similarly, in the studies by Waseem et al. and Li et al., 86.8% and 80% of females, respectively, were included, which shows an increasing trend of RA and hypothyroidism in the female gender [9,1].

Waseem et al. showed that disease activity score was higher among the patients with hypothyroidism as compared to euthyroid and hyperthyroid patients with RA. Similarly, these patients have an increased number of tender joints, but the number of swollen joints was not significantly higher in hypothyroid patients [9]. Similarly, a study by Emamifar et al. showed that 79.7% of patients in their study were females showing female predominance with hypothyroidism. Hypothyroidism was present in 30.6% of patients, which is consistent with our results. This study also stated that a significantly high disease activity score is present in the patients of RA with hypothyroidism, which is comparable to our results [2]. A study by Joshi et al. showed that hypothyroidism was present in 38.4% of patients with RA included in the study, which is comparable to our results. DAS-28, VAS score, and the tender joint count were also significantly higher in these patients, which is consistent with our results [18]. A study by Koszarny et al. showed that DAS-28 was significantly higher in patients with RA who had hypothyroidism as compared to patients who were euthyroid [19].

The limitation of this study is that we have included all patients of RA according to European League Against Rheumatism (EULAR)/ACR criteria 2010. This may have some bias as patients with the old disease may show high disease activity, which may affect the overall results.

## Conclusions

We conclude that patients of RA with concomitant hypothyroidism had increased disease activity with increased tender joints. Thyroid function tests should be included in the clinical evaluation of RA patients. The evaluation of thyroid functional status must be done during initial investigations in RA patients. This will detect thyroid disorders earlier, with early treatment initiation and possibly a better prognosis.
